# The complete chloroplast genome sequence of a medicinal orchid species *Coelogyne fimbriata* (Orchidaceae)

**DOI:** 10.1080/23802359.2020.1823264

**Published:** 2020-09-29

**Authors:** Ziyang Yue, Huixia Kao, Yu Zhang, Tiemei Wang, Shubin Dong, Jin Cheng

**Affiliations:** a College of Biological Sciences and Technology, National Engineering Laboratory for Tree Breeding, Beijing Forestry University, Beijing, China; b Beijing Advanced Innovation Center for Tree Breeding by Molecular Design, Beijing, China; c Beijing Botanical Garden, Beijing Floriculture Engineering Technology Research Centre, Beijing Laboratory of Urban and Rural Ecological Environment, Beijing, China; d School of Grassland Science, Beijing Forestry University, Beijing, China

**Keywords:** *Coelogyne fimbriata*, complete chloroplast genome, Orchidaceae, phylogenomics

## Abstract

*Coelogyne fimbriata* is an important orchid species with high medicinal value. Its complete chloroplast genome is 158,935 bp in length, which possesses the typical structure consising of a small single-copy region (SSC) of 18,743 bp, two inverted repeats (IRs) of 26,374 bp, and a large-single copy region (LSC) of 87,444 bp. The genome encodes 137 genes, including 91 protein-coding genes (PCGs), 38 tRNA genes and 8 rRNA genes. And the overall GC content is 37.40%. In addition, our phylogenetic analysis based on cp genome revealed the phylogenetic relationship between *C. fimbriata* and other 22 species in Orchidaceae.

*Coelogyne fimbriata* (Orchidaceae) is a perennial herb native to the forest edge at an altitude of 500-1200 m in southern Jiangxi, Guangdong, Hainan, Guangxi, Yunnan and Southeast Tibet, China (Chen et al. 1999). *Coelogyne fimbriata* has been reported to have important medicinal value (Chen et al. 2006; Sharma et al. 2017). However, the population resources of *C. fimbriata* continuously face decreasing due to the habitat destruction and transitional collection. Recently, Chloroplast DNA-based studies provide a remarkable breakthrough with data invaluable for studying genetic history and phylogeny (Yang et al. 2012; Givnish et al. 2015). Thus, we sequenced and characterized the plastome of *C. fimbriata* using the next-generation sequencing method to reveal the phylogenetic relationship of *C. fimbriata*.

We sampled the fresh leaf material from a plant specimen (now it is stored in the herbarium of Plant Biology Department, Beijing Forestry University, voucher No. Yachang 201901), which was collected from Leye County, Baise City, Guangxi Province, with geographic coordinates of 24.810152°N, 106.373218°E. We chose CTAB method to extract total genomic DNA (Doyle and Doyle 1987), and Illumina Novaseq 6000 platform was applied to perform 300 bp pair-end sequencing with the strategy of Illumina PE150. Plastome genome assembly was performed by Geneious (version 11, https://www.geneious.com), with the assembling algorithm of medium-low sensitivity. Due to the lack of published chloroplast genome sequences of the genus *Coelogyne* in NCBI database, clean reads were mapped to published chloroplast genome of some species (Orchidaceae): *Pleione bulbocodioides* (NC_036342), *P. chunii* (MK792342), *P. formosana* (NC_042197), *P. forrestii* (MK370035), *Changnienia amoena* (NC_045402), *Dendrobium nobile* (KX377961) and *Dendrobium parciflorum* (LC193512) as references. Filtered reads were then used for de novo assembly method with Geneious R11. The complete chloroplast sequence was annotated using PGA (Qu et al. 2019; Wang et al. 2020). The MAFFT software was implemented in multiple sequence alignment of full chloroplast sequences (Katoh and Standley 2013). Finally, we obtained a complete chloroplast genome of *Coelogyne fimbriata* and submitted it to GenBank. The accession number is MT548043.

The complete double-stranded circular chloroplast genome of *Coelogyne fimbriata* is 1,58,935 bp in length, containing a typical quadripartite structure consisting of two inverted repeats (IRs) of 26,374 bp each, separated by a large single-copy region (LSC) of 87,444 bp and a small single-copy (SSC) region of 18,743 bp. The total GC content is 37.40%. The plastome genome contains 137 functional genes, including 91 protein-coding genes (PCGs), 38 tRNA genes and 8 rRNA genes. Among them, 12 protein-coding genes (*rps16*, *atpF*, *ropC1*, *ycf3*, *rps12*, *clpP*, *petB*, *petD*, *rpl16*, *rpl2*, *ndhB*, *rps12*) and 6 tRNA genes (*trnK-UUU*, *trnG-GCC*, *trnL-UAA*, *trnV-UAC*, *trnI-GAU*, *trnA-UGC*) have introns.

We constructed the phylogenetic tree based on 22 related complete chloroplast genomes of orchids and 2 non-orchid species downloaded from GenBank. All sequences were subsequently checked and unified gene alignment in Genious R11. The maximum-likelihood-based phylogenetic tree was constructed by MEGA (version 6, https://www.megasoftware.net/) under the nucleotide substitution model of Tamura-Nei, which has been evaluated as a more practical and advantageous model in estimating the branch length of the tree, especially when the number of nucleotides is very large (Tamura and Nei 1993). The results showed that *Coelogyne fimbriata* grouped with *Pleione formosana* (NC042197) with 99% bootstrap support (1000 replicates) (Figure 1). Our phylogenetic tree shows agreements with the previous studies including morphologic and molecular identification. Many molecular-based phylogeny studies demonstrated that *Coelogyne*, *Pleione*, *Bletilla* and *Arundina* were grouped together (Burns-Balogh and Funk 1986; Givnish et al. 2015; Freudenstein and Chase 2015; Tang et al. 2017; Tan et al. 2020). Similar results were obtained by methods of the traditional morphological classification, such as *Coelogyne* and *Pleione* were belonged to the tribe of Coelogyninae (Chen et al. 1999, 2009). The cp sequence we published provides a reference for the discussion of the genetic diversity in Orchidaceae.

**Figure 1.**
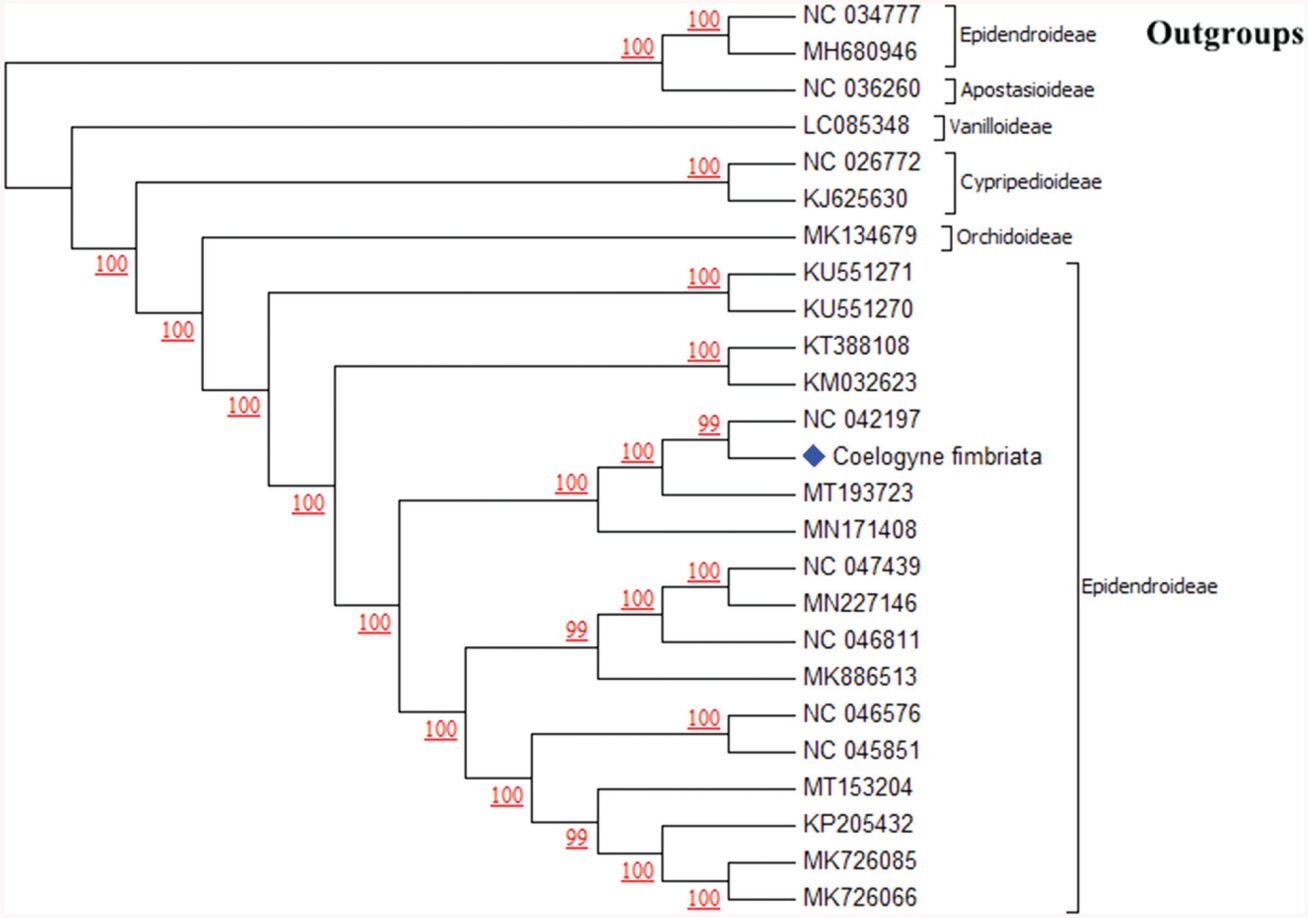
Phylogenetic tree constructed using full length plastome sequences of *Coelogyne fimbriata* and other 22 species in Orchidaceae and 2 non-orchid species. The bootstrap support values on each branch are based on Maximum likelihood methods with 1000 replicates.

## Data Availability

The basic data that support the findings of this study are openly available in NCBI (https://www.ncbi.nlm.nih.gov/) with the accession number of MT548043, or available from the corresponding author.
